# In vitro anti-HIV and antioxidant activity of *Hoodia gordonii* (Apocynaceae), a commercial plant product

**DOI:** 10.1186/s12906-016-1403-7

**Published:** 2016-10-24

**Authors:** Petrina Kapewangolo, Michael Knott, Regina E. K. Shithigona, Sylvia L. Uusiku, Martha Kandawa-Schulz

**Affiliations:** 1 Department of Chemistry and Biochemistry, Faculty of Science, University of Namibia, P/Bag 13301, Windhoek, Namibia; 2 School of Pharmacy, University of Namibia, P/Bag 13301, Windhoek, Namibia

**Keywords:** *Hoodia gordonii*, HIV RT inhibitor, HIV PR inhibitor, Antioxidant activity, Phytochemicals

## Abstract

**Background:**

*Hoodia gordonii* products are widely commercialized for anti-obesity purposes; however, minimal research is available on the other health properties demonstrated by this popular herbal plant.

**Methods:**

*H. gordonii* crude extracts (ethanol and ethyl acetate) were assayed for in vitro anti-HIV-1 protease (PR), reverse transcriptase (RT) and integrase activity. The 2,2-diphenyl-1-picrylhydrazyl (DPPH) and reducing power assays were used for the antioxidant analysis. In addition, qualitative and quantitative phytochemical analyses of the extracts were determined using standard methods.

**Results:**

*H. gordonii* extract demonstrated good inhibition against HIV RT with IC_50_ values of 73.55 ± 0.04 and 69.81 ± 9.45 μg/mL for ethanol and ethyl acetate extracts, respectively. Both extracts also demonstrated inhibitory activity against HIV PR with IC_50_ values of 97.29 ± 0.01 and 63.76 ± 9.01 μg/mL for ethanol and ethyl acetate extracts. In addition, *H. gordonii* also showed good antioxidant activity with IC_50_ values of 124.6 ± 11.3 and 126.2 ± 3.15 μg/mL obtained for ethanol and ethyl acetate extracts, respectively. The reducing power of *H. gordonii* extracts increased as the concentration increased which confirmed the presence of antioxidants (reductants) in the extracts. Phytochemical screening of *H. gordonii* revealed the presence of phenolics, alkaloids, terpenes, steroids, cardiac glycosides and tannins in the ethanolic extract, while the ethyl acetate extract only showed the presence of phenolics, cardiac glycosides and steroids. The total phenolic content was 420 ± 0.17 and 319.9 ± 0.2 mg GAE/g for the ethanol and ethyl acetate extracts, respectively. The ethanol extract, which revealed the presence of tannins, had a tannin content of 330 ± 0.2 mg TAE/g extract.

**Conclusion:**

This data suggests that *H. gordonii* has good in vitro inhibition against selected HIV-1 enzymes as well as antioxidant properties, suggesting new potential uses for this commercial plant.

## Background

*Hoodia* is a genus of succulent plants belonging to the family Apocynaceae. It is widely used now and traditionally by the San Bushmen of Southern Africa, who believe that *Hoodia* is their food, water and medicine [1, 2]. *Hoodia* species are indigenous to the Kalahari Desert of Southern Africa, including Namibia, South Africa, Angola and Botswana. One of the popular *Hoodia* species used is *Hoodia gordonii*, a desert plant traditionally used by the San people as an appetite suppressant, thirst quencher and to treat severe abdominal cramps, haemorrhoids, tuberculosis, indigestion, minor infections, hypertension and diabetes [2]. *H. gordonii* has been known by the indigenous populations of Southern Africa for a long time. For centuries this plant has been used to stave off hunger during long and tiring hunting trips or when food supplies were low [2]. Despite its popular use and commercialization, the bioactivity of *H. gordonii* has not been extensively studied.

A number of plants from the Apocynaceae family are considered to be potential sources of antioxidants which have been attributed to the high phenolic content in the phytochemical profile of some of these plants [3–5]. Antioxidants may be defined as free radical scavengers which protect living organisms from damage caused by the accumulation of free radicals. Free radicals have been implicated in various pathological conditions such as ischemia, anaemia, asthma, arthritis, inflammation, neurodegeneration, as well as speeding up the ageing process and perhaps even causing certain dementias [6–10]. Free radicals are produced by physiological and biochemical processes, or induced by environmental factors such as pollution and are capable of reacting with membrane lipids, nucleic acids, proteins and enzymes, and other small molecules which result in cellular damage [11].

Oxidative stress has also been implicated in the pathogenesis of HIV/AIDS since the virus replicates in a highly oxidized environment [12]. There is ongoing search for better or alternative treatment that could also serve as adjuvant therapy to existing anti-HIV medicines. In addition to various severe side effects, antiretroviral (ARV) drugs reportedly increase oxidative stress [13]; hence the need for antioxidants as adjuvant therapy for HIV therapy. In 2015, Tabe and colleagues administered *Hibiscus sabdariffa* (Linnaeus) juice to HIV/AIDS patients on ARV therapy and reported an increase in white blood cells compared to the control group. *H. sabdariffa* is a plant with high antioxidant capacity and is consumed as a leafy vegetable and herbal tea in many countries [14, 15]. This study investigated the antioxidant and anti-HIV potential of *H. gordonii*, a popular plant which has been commercialized as a diet suppressant to aid with weight loss [2]. This data suggests potentially new applications for this plant in the future.

## Methods

### Collection and preparation of plant materials

Dried plant material identified as *H. gordonii* was kindly donated by Farm Vredelus in July 2014. Farm Vredelus is a commercial medicinal plant farm based in Mariental, Namibia. A mechanical blender was used to grind the plant material. Plant identification was done by Silke Rugheimer at the National Herbarium of Namibia. Voucher number M1 [*H. gordonii* (Masson) Sweet ex Decne].

### Extraction

Plant material (108.3 g) was macerated at room temperature in 1 L of ethanol for 48 h. The filtrate was then concentrated under reduced pressure using a rotary evaporator and half of the residue obtained was further extracted in ethyl acetate to exclude highly polar tannins which are regarded as non-specific enzyme inhibitors [16]. The extracts obtained were dried in a fume hood and stored at room temperature until further use.

### Phytochemical analysis

Qualitative phytochemical analysis was conducted using standard procedures previously described [17, 18]. The metabolites screened for were flavonoids, phenolics, alkaloids, terpenes, steroids, cardiac glycosides, tannins and quinones. The quantitative phytochemical analysis of *H. gordonii* extracts was also carried out to determine the total phenol and tannin contents, which are amongst the most popular natural antioxidants reported in plants [19, 20].

#### Test for flavonoids

Dilute ammonia solution (5 mL) was added to a portion of the crude extract followed by addition of concentrated H_2_SO_4_. A yellow coloration observed in each extract indicated the presence of flavonoids.

#### Test for phenolics

A few drops of 5 % ferric chloride were added to extracts dissolved in distilled water. A dark green colour indicated the presence of phenolic compounds.

#### Test for alkaloids

Extracts were mixed with 2 mL of Wagner’s reagent and a reddish brown coloured precipitate indicated the presence of alkaloids.

#### Test for terpenes

The extract (5 mL) was first mixed with 2 mL of chloroform and 3 mL of concentrated H_2_SO_4_ was slowly added to form a layer. A reddish brown coloration of the interface indicated the presence of terpenes.

#### Test for steroids

0.5 mL of crude extract was mixed with 2 mL of acetic anhydride. This was followed by the subsequent addition of 2 mL H_2_SO_4_. A colour change from violet to blue or green in samples indicates the presence of steroids.

#### Test for cardiac glycosides

Exactly 5 mL of extract was treated with 2 mL of glacial acetic acid containing one drop of ferric chloride solution. The mixture was layered with 1 mL of concentrated H_2_SO_4_. A brown ring at the interface is an indication of the presence of the cardiac glycoside constituent.

#### Test for tannins

Each extract (1 mL) was mixed with 1 mL of 0.008 M Potassium ferricyanide. 0.02 M Ferric chloride in 0.1 M HCl (1 mL) was added and observed for blue-black coloration.

#### Test for quinones

Dilute NaOH was added 1 mL of crude extract. The development of a blue green or red coloration indicates the presence of quinones.

### Total phenolic content

The total phenolic content (TPC) of *H. gordonii* extracts was carried out following a method previously described [21], with modification. Extracts (0.5 g) macerated with 10 mL of 80 % ethanol were filtered and 2.5 mL of the filtrate was subsequently added to 0.25 mL of 2 M Folin–Ciocalteu reagent. The mixture was allowed to stand for 30 min and then 2 mL of 20 % sodium carbonate was added. The absorbance was measured at 650 nm using a SpectraMax M2 plate reader. A standard calibration curve (R^2^ = 0.944) was constructed using various concentrations of gallic acid (0.63, 1.25, 2.5, 5 and 10 mg/mL). TPC was expressed as milligrams (mg) of gallic acid equivalents per gram (g) of extract (mg GAE/g extract).

### Total tannin content

The total tannin content (TTC) was conducted following a procedure previously described [22], with modification. Briefly, 100 mg of the sample was macerated with 5 mL of distilled water and filtered. The filtrate (1 mL) was transferred into test tubes and mixed with 2 mL of concentrated picric acid. Absorbance was measured at 530 nm using a SpectraMax M2 plate reader. TTC was determined from extrapolation of a standard calibration curve (R^2^ = 0.966) prepared using various concentrations of tannic acid (0.63, 1.25, 2.5, 5 and 10 mg/mL). TTC was expressed as mg tannic acid equivalents per g of extract (mg TAE/g extract).

## In vitro anti-HIV assays

### HIV-1 reverse transcriptase colorimetric assay

The effect of *H. gordonii* crude extracts on HIV-1 reverse transcriptase (RT) was tested using an RT colorimetric ELISA kit from Roche Diagnostics (Mannheim, Germany). The assay was performed according to the manufacturer’s instructions. Extracts were tested at six different concentrations (50, 100, 200, 400, 800 and 1000 μg/mL). The enzyme was incubated for 1 h with extract at 37 °C. Subsequent 1 h incubations included addition of an antibody conjugated to peroxidase that binds to the digoxigenin-labeled DNA. In the final step, the ABTS substrate solution was cleaved by the peroxidase enzyme, producing a coloured reaction product. Doxorubicin, a known HIV-1 RT inhibitor was used as a positive control. The absorbance of the samples was read at 405 nm using a SpectraMax M2 plate reader.

### HIV-1 integrase assay

The Xpress HIV-1 Integrase Assay Kit (Express Biotech International, USA) was used to measure the inhibitory effects of *H. gordonii* extracts (0.1, 0.2 and 0.4 mg/mL) on HIV-1 integrase activity. Streptavidin coated 96-well plates were coated with a double-stranded HIV-1 LTR U5 donor substrate oligonucleotide containing an end-labelled biotin. Full-length recombinant HIV-1 integrase protein was then loaded onto the oligo substrate. *H. gordonii* extracts or sodium azide (standard control) was added to the reaction plates together with a double-stranded target substrate (TS) oligo containing 3′-end modifications. The horseradish peroxidase (HRP)-labelled antibody was directed against the TS 3′-end modification and the absorbance due to the HRP antibody– tetramethylbenzidine peroxidase substrate reaction was measured at 450 nm using a SpectraMax M2 plate reader.

### HIV-1 protease fluorogenic assay

A SensoLyte 490 HIV-1 Protease (PR) kit from AnaSpec (San Jose, CA, USA), was used to assay *H. gordonii* extracts against HIV-1 PR. Due to the limited number of reactions of the kit, samples were tested at five concentrations, namely; 25, 50, 100, 200 and 400 μg/mL. Acetyl pepstatin (AP) was used as a known standard for HIV-1 PR inhibition. Briefly, test samples were incubated at room temperature with HIV-1 PR enzyme and substrate for 45 min. Stop solution (50 μl) was added to each reaction then the fluorescence intensity was measured at Excitation/Emission = 340/490 nm using a SpectraMax M2 plate reader.

## Antioxidant activity

### 2, 2-Diphenyl-1-picryl-hydrazyl radical scavenging assay

2, 2-Diphenyl-1-picryl-hydrazyl (DPPH: Sigma-Aldrich, Germany) is a stable free radical with a purple colour and upon scavenging, these free radicals turn to yellow. The free radical scavenging activity of the extract was evaluated using a modified method previously described [16]. Various concentrations of *H. gordonii* extracts were mixed with 90 μM DPPH. Since DPPH is light sensitive, incubation was done in the dark at room temperature for 30 min. The absorbance of the resulting solution was measured using a plate reader at 520 nm. Vitamin C (ascorbic acid) was used as a positive control.

### Reducing power assay

The ability of *H. gordonii* extracts to reduce iron (III) was determined according to the Kadri’s method [23] with some modifications. Different concentrations of extracts were mixed with 2.5 mL of phosphate buffer (1 M, pH 6.6) and 2.5 mL of 1 % potassium ferricyanide. The mixture was incubated at 40 °C for 20 min. After incubation, 2.5 mL of 10 % trichloroacetic acid was added and centrifuged for 10 min at 3000 rpm. To 2.5 mL of this reaction mixture (upper layer), 0.5 mL of ferric chloride and 2.5 mL of water was added. Ascorbic acid was used as a reference standard. The absorbance was measured at 700 nm spectrophotometrically.

### Data analysis

The data is presented as mean plus or minus standard error of the mean (M ± SEM). The 50 % inhibitory concentrations (IC_50_ values) for enzymes and DPPH assays were computed using Graphpad Prism 5 software (Graphpad Software Inc. California, USA).

## Results

### Qualitative and quantitative phytochemical analysis of *H. gordonii* extracts

Phytochemical results revealed the presence of a number of phytochemicals including phenolic components, alkaloids, terpenes, steroids and cardiac glycosides as shown in Table 1. It was noted that quinones and flavonoids were absent from *H. gordonii* extracts.

**Table 1:** Phytochemical analysis of *H. gordonii* using ethanol and ethyl acetate extracts

Phytochemical	Results	
	Ethanol	Ethyl acetate
Flavonoids	–	–
Phenolics	+	+
Alkaloids	+	–
Terpenes	+	–
Steroids	+	+
Cardiac glycosides	+	+
Tannins	+	–
Quinones	–	–

(+): Indicates the presence of chemical constituents; (−): Indicates the absence of chemical constituents

### Total phenolic and tannin contents

The amount of total phenolic content of the extracts was found to be 420 ± 0.17 and 319.9 ± 0.2 mg GAE/g for ethanol and ethyl acetate extracts, respectively. Due to the absence of tannins in the ethyl acetate extract, as revealed by the phytochemical screening, the total tannin content was only determined for the ethanol extract of *H. gordonii* and the amount obtained was 330 ± 0.2 mg TAE/g extract. These results indicate that *H. gordonii* could be a rich source of tannins and phenolic compounds.

### In vitro anti-HIV potential of *H. gordonii*

**Fig. 1:**
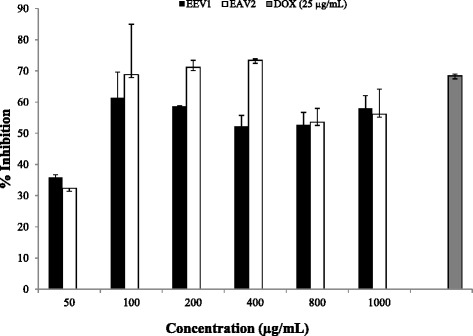
In vitro anti-HIV RT activity of *H. gordonii* ethanol (EEV1) and ethyl acetate extract (EAV2). Doxorubicin was used as a positive control. IC_50_ values were 73.55 ± 0.04 and 69.81 ± 9.45 μg/mL for ethanol and ethyl acetate extract, respectively

Both *H. gordonii* ethanol and ethyl acetate extracts exhibited good inhibition in a dose-dependent manner (Fig. 1) against HIV-1 Reverse transcriptase (RT) with IC_50_ values of 73.55 ± 0.04 and 69.81 ± 9.45 μg/mL, respectively (Table 2). Doxorubicin, a known RT inhibitor [16], was used as a positive control and inhibited HIV RT by 68 % at 25 μg/mL (IC_50_ < 25 μg/mL). Both extracts also demonstrated inhibitory activity against HIV protease (PR) with IC_50_ values of 97.29 ± 0.01 and 63.76 ± 9.01 μg/mL for ethanol and ethyl acetate extracts, respectively. Acetyl pepstatin was used as a known PR inhibitor and inhibited HIV PR by as much as 82 % at 50 μg/mL (IC_50_ < 50 μg/mL). Both ethanol and ethyl acetate extracts had weak inhibition against HIV-1 integrase (IN) with <50 % inhibition at the highest concentration tested of 400 μg/mL. Sodium azide was used as a positive control compound for IN inhibition.

**Table 2:** IC_50_ ± S.D values of crude *H. gordonii* ethyl acetate and ethanolic extracts against HIV-1 RT, PR and IN activity

Sample	HIV RT (μg/mL)	HIV PR (μg/mL)	HIV IN (μg/mL)
Ethanol extract	73.55 ± 0.04	97.29 ± 0.01	>400
Ethyl acetate extract	69.81 ± 9.45	63.76 ± 9.01	>400
Doxorubicin^a^	<25	–	–
Acetyl pepstatin^b^	–	<50	–
Sodium azide^c^	–	–	<50

^a^A known HIV reverse transcriptase inhibitor

^b^A known HIV protease inhibitor

^c^A known HIV integrase inhibitor

### DPPH (2, 2-Diphenyl-1-picryl-hydrazyl) assay

DPPH radical scavenging is one of the most widely used methods for assaying the antioxidant activity of compounds and plant extracts. The DPPH method is easy, rapid and sensitive; the DPPH free radical is stable at room temperature and accepts an electron or hydrogen to become a stable diamagnetic molecule [24].

**Fig. 2:**
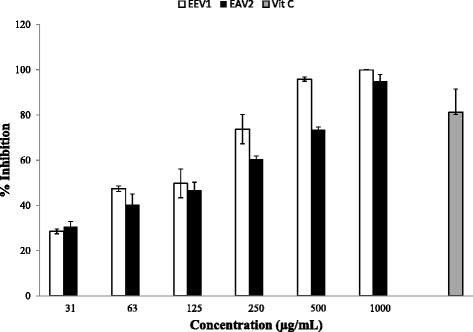
DPPH free radical scavenging activity of ethanol (EEV1) and ethyl acetate (EAV2) extracts of *H. gordonii*. The IC_50_ values for the ethanol and ethyl acetate extracts were 124.6 ± 11.3 and 126.2 ± 3.15 μg/mL, respectively. Ascorbic acid (Vit C) was used as a positive control (IC_50_ < 50 μg/mL)

The investigated *H. gordonii* extracts (ethanol and ethyl acetate) exhibited good antioxidant properties in a concentration dependent manner (Fig. 2). The IC_50_ values of the ethanol and ethyl acetate extracts were 124.6 ± 11.3 and 126.2 ± 3.15 μg/mL, respectively. DPPH scavenging activity of the ethanol extract was slightly higher than that of ethyl acetate extract. Ascorbic acid was used as a standard antioxidant control (IC_50_ < 50 μg/mL) because of its ability to scavenge free radicals.

### Reducing power assay

**Fig. 3:**
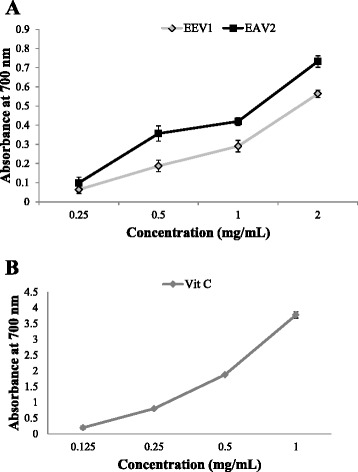
Reducing power of *H. gordonii* ethanol (EEV1) and ethyl acetate (EAV2) extracts at various concentrations. Each value is expressed as mean ± standard deviation (*n* = 3). (**a**) The reducing power of *H. gordonii* extracts increased with increased concentration. (**b**) Ascorbic acid (Vit C) was used as a standard control

Determination of the ferric reducing power is a simple direct test of antioxidant capacity. As illustrated in Fig. 3, the conversion of Fe^3+^ to Fe^2+^ in the presence of *H. gordonii* extracts could be measured as their reductive ability. The presence of reductants such as antioxidants in *H. gordonii* extracts caused the reduction of the Fe^3+^/ferricyanide complex to a ferrous form. The results (Fig. 3a) showed a concentration-dependent significant increase (*P* < 0.05) in the reductive ability of *H. gordonii* ethanol and ethyl acetate extracts. The results were compared to ascorbic acid, a standard control (Fig. 3b).

## Discussion

A number of glycosides have been isolated from *H. gordonii* [2] and amongst these glycosides is the popular P57 glycoside attributed to the appetite suppressant properties of *H. gordonii*. The attention on *H. gordonii* species was elicited by the discovery of hunger suppressing glycosides. Despite its popular use, minimal reports are available on biological studies conducted on *H. gordonii*. However, the safety profile of *H. gordonii* extracts has already been determined in a number of in vivo studies [25].

The only antioxidant study conducted on *H. gordonii* was on glycosides isolated from the plant which did not demonstrate antioxidant property [2]. The presence of other phytochemicals in the crude extracts such as phenolics, alkaloids, tannins and terpenes could be attributed to the antioxidant potential observed in this study. The *H. gordonii* ethanol extract exhibited the highest reducing activity and these results were in agreement with the high DPPH scavenging activity observed in ethanol extracts. The total phenolic content of the ethanol extract was relatively high compared to that of the ethyl acetate extract and the presence of tannins in the ethanol extract but not in ethyl acetate extract could all be responsible for the high antioxidant potential observed in the ethanol extract. Phenolic compounds and tannins are widely reported as natural antioxidants [19, 20] and the present study revealed that *H. gordonii* could be a potential source of useful natural antioxidants. *H. gordonii* is among the most popular anti-obesity products on the market [26]. Obesity is a chronic disease and amongst other morbidities, it is associated with an increase in oxidative stress [27]. The role of oxidative stress in the pathogenesis of various ailments, such as psychiatric, inflammatory and infectious diseases has been well documented [6, 9, 12, 28]. The antioxidant activity observed in the present study could contribute to the scavenging of accumulated free radicals in mostly obese individuals that consume *H. gordonii* products.

In addition to being associated with obesity, oxidative stress has also been linked to the progression of HIV [29] which is supported by a study that reported the promotion of HIV replication by oxidizing agents as compared to antioxidants [30]. Before the present study, there was no literature reporting on the in vitro anti-HIV-1 properties of *H. gordonii*. The extracts demonstrated good inhibition against HIV-1 reverse transcriptase and protease which are two of the three HIV enzymes that play a major role in the replication of the virus in host cells. Current HIV therapy targets various steps of the HIV life cycle, which includes HIV enzymes [31]. However, this antiretroviral therapy is often limited by adverse side effects leading to patients discontinuing treatment and in the process contributing to the development of HIV drug resistant strains [31]. The search for better HIV therapy is ongoing and the in vitro anti-HIV data from the present study is a valuable contribution towards this search. *H. gordonii* is already regarded as a complementary and alternative medicine for the treatment of obesity [26]. Further in vivo validation of this research could support the use of this commercial product as a supplement for HIV therapy as well as a natural antioxidant.

## Conclusion

The in vitro anti-HIV and antioxidant data obtained in this study suggests new potential uses of *H. gordonii*, which is currently commercialized and mainly used as an anti-obesity supplement. The chemistry of *H. gordonii* has been reported as well as the isolation and characterization of the glycosidic compounds. However, based on the results of this work, future investigations will also research the isolation and characterization of *H. gordonii* compounds which are responsible for the above mentioned in vitro anti-HIV-1 and antioxidant activity.

## Abbreviations

AIDS: Acquired immunodeficiency syndrome
AP: Acetyl pepstatin
ARV: Antiretroviral
DOX: Doxorubicin
DPPH: 2,2-diphenyl-1-picrylhydrazyl
EAV2: Ethyl acetate extract
EEV1: Ethanol extract
HIV: Human immunodeficiency virus
HRP: Horseradish peroxidase
IC_50_: 50 % inhibitory concentration
IN: Integrase
LTR: Long terminal repeat
PR: Protease
RT: Reverse transcriptase
TS: Target substrate.
